# Phylogenomic analyses support the position of turtles as the sister group of birds and crocodiles (Archosauria)

**DOI:** 10.1186/1741-7007-10-65

**Published:** 2012-07-27

**Authors:** Ylenia Chiari, Vincent Cahais, Nicolas Galtier, Frédéric Delsuc

**Affiliations:** 1 Institut des Sciences de l'Evolution, UMR5554-CNRS-IRD, Université Montpellier 2, Montpellier, France; 2 CIBIO, Centro de Investigação em Biodiversidade e Recursos Genéticos, Campus Agrário de Vairão, 4485-661 Vairão, Portugal

## Abstract

**Background:**

The morphological peculiarities of turtles have, for a long time, impeded their accurate placement in the phylogeny of amniotes. Molecular data used to address this major evolutionary question have so far been limited to a handful of markers and/or taxa. These studies have supported conflicting topologies, positioning turtles as either the sister group to all other reptiles, to lepidosaurs (tuatara, lizards and snakes), to archosaurs (birds and crocodiles), or to crocodilians. Genome-scale data have been shown to be useful in resolving other debated phylogenies, but no such adequate dataset is yet available for amniotes.

**Results:**

In this study, we used next-generation sequencing to obtain seven new transcriptomes from the blood, liver, or jaws of four turtles, a caiman, a lizard, and a lungfish. We used a phylogenomic dataset based on 248 nuclear genes (187,026 nucleotide sites) for 16 vertebrate taxa to resolve the origins of turtles. Maximum likelihood and Bayesian concatenation analyses and species tree approaches performed under the most realistic models of the nucleotide and amino acid substitution processes unambiguously support turtles as a sister group to birds and crocodiles. The use of more simplistic models of nucleotide substitution for both concatenation and species tree reconstruction methods leads to the artefactual grouping of turtles and crocodiles, most likely because of substitution saturation at third codon positions. Relaxed molecular clock methods estimate the divergence between turtles and archosaurs around 255 million years ago. The most recent common ancestor of living turtles, corresponding to the split between Pleurodira and Cryptodira, is estimated to have occurred around 157 million years ago, in the Upper Jurassic period. This is a more recent estimate than previously reported, and questions the interpretation of controversial Lower Jurassic fossils as being part of the extant turtles radiation.

**Conclusions:**

These results provide a phylogenetic framework and timescale with which to interpret the evolution of the peculiar morphological, developmental, and molecular features of turtles within the amniotes.

## Background

Turtles (order Testudines) represent one of the most anatomically peculiar vertebrate groups. Their highly derived morphology relative to other vertebrates arose through profound structural changes associated with the origin of the shell [1]. Turtles have been described as having a conspicuously modified reptile body plan, and termed 'hopeful monsters', representing a successful phenotypic mutant with the potential to establish a new evolutionary lineage [2-7]. These morphological adaptations make it difficult to compare turtles with other organisms and to establish the polarity of numerous anatomical characters as either being ancestral or derived. Because turtles represent one of the major groups of amniotes, resolving their phylogenetic position would fill an important gap in the evolutionary history of vertebrates, and contribute to the understanding of how such a key innovation as the turtle shell originated and which underlying genes are involved in its development [8].

The phylogenetic relationships of turtles within the amniotes have puzzled scientists for more than a century. Turtles have been classified both as basal to all other reptiles (including birds) and as nested within them, implying two radically different perspectives from which to interpret the evolution of morphological, developmental, molecular, or ecological data. The classic view [9,10] places turtles as the sister group to all other reptiles, mostly on the basis of the lack of temporal fenestration in the skull, a character considered as being ancestral for Reptilia. This view reflects the traditional dichotomy of reptiles as Anapsida (lacking temporal fenestration) or Diapsida (with two temporal fenestrations). However, cladistic studies of morphological datasets have generated conflicting results, supporting both an anapsid [11-13] and a diapsid [14-16] affinity of turtles, depending on the fossil sampling considered and the morphological matrices used. Harris *et al. *[17] showed how the morphological characters used to assess the phylogenetic placement of the turtles within the tree of amniotes can lead to conflicting signals, and suggested, given these difficulties, that the answer to this long-standing controversy would most probably come from molecular data.

However, the use of molecular data has not yet settled the debate, as it has also provided somewhat conflicting results. In the large number of publications on the topic over the past decade, turtles have been grouped with Archosauria (birds and crocodiles) in most studies [18-22], but have also been grouped with crocodiles [23-26] or sometimes with Lepidosauria (tuatara, lizards, and snakes) [27]. The causes of the conflicting signals and/or lack of resolution obtained in most studies have been attributed to the limited number of genes considered, poor taxon sampling, substitution rate heterogeneity among genes and among taxa, and saturation or selection occurring at some of the markers [28]. Moreover, statistical tests performed to evaluate alternative topologies based on these early molecular sequence datasets usually failed to reach significance, probably because of the reduced number of genes included, but also possibly because of heterogeneity in gene trees.

With the advent of genomic data, the comparative datasets increased in size, but the issue of turtle phylogeny remained unresolved. The first investigation of genome structure and composition in reptiles identified a similarity in genomic signatures between turtles and crocodiles [29]. A recent multigene study offered the first convincing support for the grouping of turtles and archosaurs [30], but this result was contradicted by a newer study based on the distribution of microRNAs (miRNA), which strongly suggested an alternative turtles plus lizards clade [31]. Finally, a recent phylogenomic study based on reptile transcriptomic data did not find compelling support to distinguish between turtles plus crocodiles and turtles plus archosaurs, despite including a large number of genes [32]. In that study, the analysis of the largest dataset strongly supported a topology with the turtle as the sister group to the crocodile, whereas analyses after removing potential paralogs favoured a turtle plus archosaurs clade, albeit with reduced statistical support [32].

In the present study, we used a phylogenomic approach [33] to resolve the position of the turtles within the amniotes, and estimated the time of their origin using a dataset comprising 248 nuclear protein-coding genes for 16 vertebrates. We applied phylogenetic reconstruction methods and models of sequence evolution, explicitly accounting for substitution rate heterogeneities among taxa and among genes, and maximum likelihood (ML) species tree analyses accounting for gene tree discordance. We also used various relaxed molecular clock Bayesian approaches to reconstruct a timescale for the evolutionary history of the amniotes.

## Results

### Phylogenetic results

**Figure 1:**
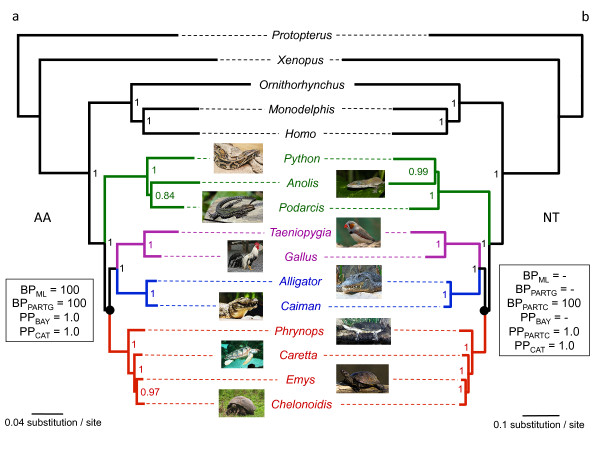
**Phylogenetic relationships of amniotes as inferred from analyses of the 248-gene dataset**. **(a) **Bayesian consensus topology obtained from analyses of the amino-acid dataset (62,342 sites) under the CAT-GTR + G4 mixture model. **(b) **Bayesian consensus topology obtained from analyses of the complete nucleotide dataset (187,026 sites) under the CAT-GTR + G4 mixture model. The nodal values indicate the clade Bayesian posterior probability (PP). Statistical support values obtained with different methods, models and data partitions detailed in Table 1 are reported in boxes for turtles plus archosaurs. Note the relative incongruence between the two trees concerning the position of *Python*. All pictures are from Wikimedia Commons, except for *Chelonoidis* from Y. Chiari. Please note also that the taxonomy of Galapagos turtles being currently revised, the appropriate species name for the *Chelonoidis* specimen included here might be *Chelonoidis* sp.

Our phylogenomic dataset provides strong support for the phylogenetic position of turtles as a sister group to Archosauria within Amniota based on concatenation analyses (Figure 1). All of our Bayesian and ML analyses of the concatenated amino-acid dataset recovered this topology with maximal ML bootstrap support (BP) and Bayesian posterior probabilities (PP) irrespective of the model used (BP_ML _= 100; BP_PARTG _= 100; PP_BAY _= 1.0; PP_CAT _= 1.0) (Figure 1a; Table 1). The same result was obtained from analyses of the complete nucleotide dataset with ML and Bayesian analyses when a mixed model partitioned by codon was applied (BP_PARTC _= 100; PP_PARTC _= 1.0), and in Bayesian analyses conducted under the site-heterogeneous CAT-GTR + G4 mixture model (PP_CAT _= 1.0) (Figure 1b; Table 1). Conversely, ML and Bayesian phylogenetic reconstructions from the complete nucleotide dataset using a single site-homogeneous GTR + G model for the whole concatenation (BP_ML _= 76; PP_BAY _= 1.0), and a mixed model partitioned by gene (BP_PARTG _= 54) tended to support an alternative topology grouping turtles with crocodilians (Table 1).

**Table 1:** Statistical support for the phylogenetic position of turtles based on the various reconstruction methods, substitution models, and data partitions.

	Amino acids	Nucleotides		
	
	All positions	All positions	Positions 1 + 2	Positions 3
**Total sites**	62,342	187,026	124,684	62,342
**Constant sites**	41,170 (66.0%)	99,638 (53.3%)	92,128 (73.9%)	7,510 (11.2%)
**Informative sites**	8,749 (14.0%)	54,880 (29.3%)	14,009 (11.2%)	40,871 (65.6%)
**RaxML **LG + G / GTR + G	Turtles + Archosaurs BP_ML _= 100	Turtles + Crocodiles BP_ML _= 76	Turtles + Archosaurs BP_ML _= 100	Turtles + Crocodiles BP_ML _= 100
**RaxML **GTR + G partitioned by gene	Turtles + Archosaurs BP_PARTG _= 100	Turtles + Crocodiles BP_PARTG _= 54	-	-
**RaxML **GTR + G partitioned by codon	-	Turtles + Archosaurs BP_PARTC _= 100	-	-
**MrBayes **WAG + G / GTR + G	Turtles + Archosaurs PP_BAY _= 1.0	Turtles + Crocodiles PP_BAY _= 1.0	Turtles + Archosaurs PP_BAY _= 1.0	Turtles + Crocodiles PP_BAY _= 1.0
**MrBayes **GTR + G partitioned by codon	-	Turtles + Archosaurs PP_PARTC _= 1.0	-	-
**PhyloBayes **CAT-GTR + G	Turtles + Archosaurs PP_CAT _= 1.0	Turtles + Archosaurs PP_CAT _= 1.0	Turtles + Archosaurs PP_CAT _= 1.0	Turtles + Archosaurs PP_CAT _= 1.0

Likelihood-based comparisons of partitioned models based on the Akaike information criterion (AIC) showed that partitioning by codon position using the GTR + G model was by far the best partition scheme (AIC_CONCAT _= 2,109,010; AIC_ByGene _= 2,082,688; AIC_ByCodon _= 2,008,142). The fact that only the suboptimal and poorly fitting models supported a turtles + crocodilians relationship suggests that this topology is a phylogenetic reconstruction artefact, most likely the result of the inability of these models to account efficiently for site-specific heterogeneities in the substitution process. The better fit offered by the codon position partition scheme over the gene partition scheme indicates that the main source of heterogeneity lies in the codon positions, most probably because of multiple substitutions accumulating at third codon positions.

**Figure 2:**
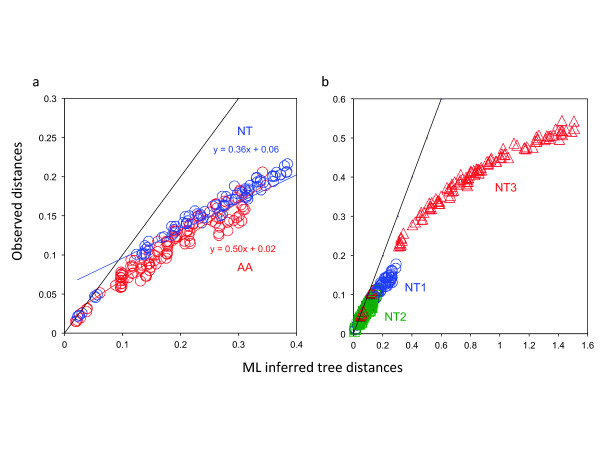
**Analyses of substitution saturation at each codon position**. Maximum likelihood saturation plots [34] were compared **(a) **between the complete amino-acid and nucleotide datasets, and **(b) **between the codon positions of the complete nucleotide dataset. The observed pairwise distances between the 16 taxa were directly computed from sequence alignments, and the corresponding inferred pairwise tree distances calculated from branch lengths of the ML topology. The Y = × line marks the theoretical limit where the number of observed substitutions equals the number of inferred substitutions. The slope of the linear regression indicates the amount of substitution saturation; the smaller the slope, the greater the number of inferred multiple substitutions.

Comparisons of ML-based saturation plots [34] between the amino-acid and the complete nucleotide datasets (Figure 2a) did not reveal clear evidence for global substitutional saturation of the complete nucleotide dataset relative to the amino-acid dataset, despite a slightly lower slope (0.36 versus 0.50, respectively). However, as expected in protein-coding genes conserved at this level of divergence, substitutional saturation was particularly pronounced at the third codon positions (Figure 2b). In cases in which the substitutional saturation of third codon positions was particularly high, excluding this third codon position partition from the dataset would be expected to result in less biased phylogenetic reconstructions. In agreement with this prediction, all ML and Bayesian reconstructions performed on the nucleotide dataset after exclusion of third codon positions provide unambiguous support (BP_ML _= 100; PP_BAY _= 1.0; PP_CAT _= 1.0) for regrouping turtles and archosaurs (Table 1). Conversely, ML and Bayesian analyses of concatenated third codon positions using a single GTR + G model returned maximal support (BP_ML _= 100; PP_BAY _= 1.0) for the topology clustering turtles with crocodilians (Table 1). Only the CAT-GTR + G4 mixture model seemed to be able to deal efficiently with the saturated third codon positions dataset by strongly supporting the turtles + archosaurs clade (PP_CAT _= 1.0). These analyses indicate that substitutional saturation at third codon positions is so strong in this phylogenomic dataset that it is able to impede phylogenetic reconstruction when inappropriate models of sequence evolution are used.

Statistical tests between competing topologies confirmed the above results (Table 2). The approximately unbiased (AU) likelihood-based test showed that all proposed alternative hypotheses to the sister group relationship of turtles with archosaurs were rejected based on the amino-acid dataset, irrespective of the model used. In concordance with the results of saturation analyses, the complete nucleotide dataset did not distinguish statistically between the competing alternatives of turtles plus archosaurs and turtles plus crocodilians. These more equivocal and method-dependent results, when nucleotide sequences were used, are suggestive of conflicting phylogenetic signals between codon positions. However, the alternative topologies proposing turtles as the sister group to other reptiles (including birds), and grouping turtles with squamates (lizards and snakes) received no support from our data.

**Table 2:** Results of the approximately unbiased likelihood-based statistical test for comparing alternative topologies using different data types and partitions.

	Amino acids		Nucleotides		
	
Topologies	Concatenated	Partitioned by gene	Concatenated	Partitioned by gene	Partitioned by codon
Turtles + archosaurs	best	best	0.26	0.85	0.91
Turtles + crocodilians	< 0.001*	< 0.001*	0.74	0.15	0.09
Turtles + squamates	< 0.001*	< 0.001*	< 0.001*	< 0.001*	< 0.001*
Turtles + other reptiles	< 0.001*	< 0.001*	< 0.001*	< 0.001*	< 0.001*

*Significant.

**Figure 3:**
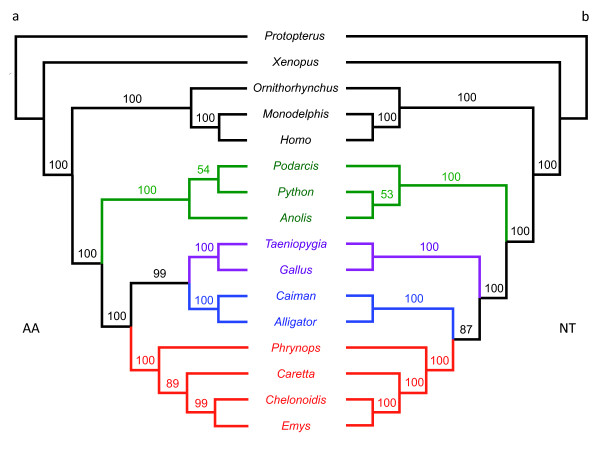
**Species trees inferred from the 248 individual maximum likelihood (ML) gene trees using a pseudo-ML approach**. Maximum pseudo-likelihood for estimating species trees (MP-EST) bootstrap consensus species tree obtained for **(a) **the amino-acid and **(b) **the nucleotide dataset. **(a) **This consensus tree was computed from the species trees estimated by the MP-EST method for 100 bootstrap datasets of the 248 ML gene trees inferred under the LG + G8 model. **(b) **This consensus tree was computed from the species trees estimated by the MP-EST method for 100 bootstrap datasets of the 248 ML gene trees inferred under the GTR + G8 model. Values at nodes indicate bootstrap percentages obtained with 100 replicates. Note the strong incongruence between the two species trees concerning the position of turtles.

Finally, given the fact that the internal branch lengths connecting the main reptiles lineages seemed to be relatively short in trees obtained from concatenated analyses (Figure 1), we also explored the potential influence of the underlying gene-tree heterogeneity created by deep coalescence events, which might lead to statistical inconsistency of concatenation-based methods in the anomaly zone [35,36]. The results obtained using the maximum pseudo-likelihood for estimating species trees (MP-EST) approach showed high consistency with the results of our concatenation-based analyses (Figure 3). Indeed, the species tree reconstructed from the amino-acid ML gene trees unambiguously supported (BP = 100) the grouping of turtles and archosaurs (Figure 3a), whereas the species tree based on nucleotide ML gene trees supported (BP = 87) a conflicting turtles plus crocodilians clade (Figure 3b), as previously shown in concatenation-based analyses using suboptimal models of sequence evolution. In fact, only six amino-acid and three nucleotide ML gene trees were fully compatible with their corresponding species trees. These figures illustrate the large extent of gene-tree heterogeneity in this dataset, which probably reflects the large effect of stochastic error on individual gene-tree inference. We interpret these congruent results between concatenation and species tree inference as good evidence that the source of the statistical inconsistency resulting in the grouping of turtles with crocodiles does not come from potential discordances between gene trees and the species tree, but rather from the influence of substitutional saturation of third codon positions in individual gene-tree inference.

### Molecular dating results

**Figure 4:**
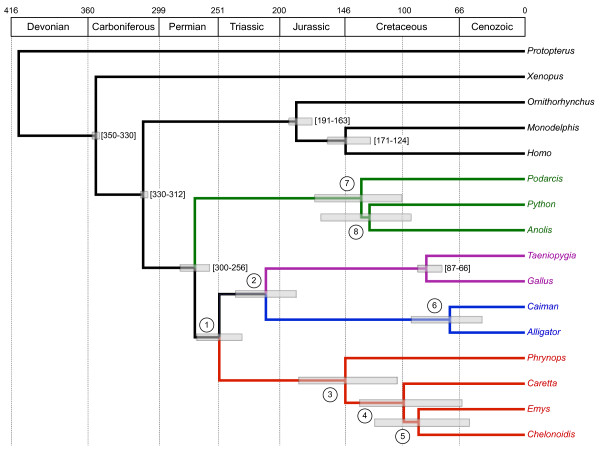
**Bayesian relaxed molecular clock time scale**. Chronogram obtained from the analysis of the nucleotide dataset using the CAT-GTR + G mixture model. Numbers in circles at nodes refer to lines of Table 3, and squared boxes represent 95% credibility intervals. Numbers between brackets represent the six calibration constraints implemented as soft bounds. Absolute ages of the geological periods follow Gradstein and Hogg [91].

Detailed results from the molecular dating analyses performed under auto-correlated models of molecular clock relaxation are presented in Table 3. Divergence date estimates varied depending on the methods and datasets used, but were nevertheless consistent between the two programs we used (MCMCTree and PhyloBayes). We generally found more consistency with published estimates for the results obtained with PhyloBayes under the CAT-GTR + G site-heterogeneous mixture model (Table 3) than for the results obtained with the site-homogeneous LG + G / WAG + G and GTR + G models. Our analyses based on the CAT-GTR + G model placed the divergence between turtles and archosaurs around the Permian-Triassic boundary at a mean of 255 Mya (range 274 to 233 Mya), the separation of crocodilians and birds in the Upper Triassic with a mean of 219 Mya (249 to 186 Mya), and the most recent common ancestor (MRCA) of living turtles (corresponding to the separation between Pleurodira and Cryptodira) in the Upper Jurassic with a mean of 157 Mya (207 to 104 Mya) depending on whether amino acids or nucleotides are considered (Table 3). The chronogram obtained from the analysis of the nucleotide dataset using the CAT-GTR + G model is shown in Figure 4.

**Table 3:** Detailed results of Bayesian relaxed molecular clock analyses obtained under different auto-correlated models for the eight unconstrained nodes^a^

	Nucleotides			Amino acids			
	
	MCMCTree WAG + G	PhyloBayes LG + G	PhyloBayes CAT-GTR + G	MCMCTree GTR + G	PhyloBayes GTR + G	PhyloBayes CAT-GTR + G	TimeTree mean/median^b^
Turtles and archosaurs MRCA	229 (200 to 253)	228 (208 to 252)	249 (233 to 270)	228 (188 to 256)	225 (212 to 240)	258 (238 to 274)	244/265
Archosaurs MRCA	200 (166 to 231)	188 (162 to 217)	211 (186 to 236)	209 (168 to 241)	201 (180 to 221)	226 (199 to 249)	238/245
Turtles MRCA	121 (63 to 183)	126 (85 to 166)	147 (104 to 185)	107 (53 to 183)	133 (93 to 171)	167 (120 to 207)	207/211
*Caretta*/*Emys *+ *Cheloinidis*	77 (40 to 127)	83 (47 to 117)	99 (64 to 139)	69 (34 to 129)	90 (52 to 125)	115 (71 to 154)	97/99
*Emys*/*Chelonoidis*	65 (25 to 116)	71 (38 to 102)	87 (54 to 124)	52 (16 to 112)	74 (39 to 106)	95 (56 to 131)	70/70
*Caiman*/*Alligator*	72 (15 to 156)	54 (33 to 86)	61 (35 to 93)	60 (11 to 160)	39 (21 to 66)	46 (24 to 87)	70/72
*Podarcis*/*Python + Anolis*	171 (119 to 218)	150 (116 to 183)	134 (100 to 172)	167 (108 to 219)	150 (122 to 181)	119 (90 to 151)	190/178
*Python*/*Anolis*	147 (81 to 198)	136 (98 to 71)	127 (93 to 167)	141 (58 to 199)	135 (101 to 161)	105 (74 to 136)	171/163

Abbreviations: MRCA, most recent common ancestor

**^a^**Values are mean (95% Credibility interval).

**^b^**Values taken from the TimeTree database [92].

Strikingly different results were obtained when using uncorrelated models of clock relaxation (see Additional file 1). Again, dating estimates were fairly consistent between the different program implementations (BEAST, MCMCTree, and PhyloBayes), but using uncorrelated rate models generally led to much smaller age estimates than the ones obtained under auto-correlated rate models. For example, using uncorrelated models, the MRCA of living turtles was estimated to be half the age of that found with auto-correlated models, with mean estimates ranging from 81 to 64 Mya versus 167 to 107 Mya, respectively. Other estimates, such as the caiman/alligator divergence, were reduced by two-thirds, resulting in unreasonably recent estimations relative to the TimeTree values (see Additional file 1).

## Discussion

### Phylogenomics and the position of turtles

Previous phylogenetic investigations of amniote phylogeny have failed to reach a clear consensus on the phylogenetic position of turtles, as the various studies have often produced ambiguous and sometimes conflicting results. The causes for this probably stem from the intrinsic difficulty of this phylogenetic problem, which involves ancient divergences. Most of the previous molecular studies addressing this question used either small datasets based on a few nuclear genes [19,24] or genetically linked mitochondrial genes [22,23,37]. In general, phylogenetic analyses based on using mitochondrial data tended to recover a sister group relationship between turtles and Archosauria [20-22,37,38], whereas some of the nuclear data favoured a clade of turtles with crocodiles [23,24,26]. The only exception to this pattern is the study by Iwabe *et al. *[19], who reported statistical support for the turtles plus archosaurs clade, but this was based on only two nuclear genes and a minimal taxon sampling.

Resolving the branching patterns of the major lineages of amniotes requires gathering a considerable amount of informative sequence data from independent markers with adequate taxon sampling. Shen *et al. *[30] recently investigated this question using 23 (mostly nuclear) markers for 28 vertebrates, and estimated that with their taxon sampling, a total sequence length of more than 13,000 nucleotides from independent nuclear markers is necessary to provide significant statistical support for resolving the controversial relationships between turtles, birds, and crocodilians. Our phylogenomic results, based on 248 nuclear markers, corroborate their predictions about the challenge represented by resolving this phylogenetic question, and add support to the sister group relationship between turtles and archosaurs (birds plus crocodilians). Furthermore, our statistical analyses reject any alternative hypotheses to the sister group relationship of turtles to Archosauria (Table 2), thus advancing the resolution of this long-standing controversial issue of vertebrate evolutionary history.

However, as illustrated by the occurrence of conflicting signals in our phylogenetic analyses, the phylogenomic approach is not immune to statistical inconsistency [39], as highlighted here in the cases of ML and Bayesian analyses of nucleotide datasets under a single concatenated GTR + G model, and in species tree inference from nucleotide gene trees, which showed inconsistencies that are most likely due to saturation at third codon positions. The fact that turtles group with crocodilians in concatenation analyses is probably due to a long-branch attraction (LBA) artefact causing the faster evolving squamates to be attracted towards mammals and the outgroups (see Additional file 2). The same grouping of turtles + crocodiles was also retrieved with strong support by Tzika *et al. *[32] based on ML and Bayesian analyses of amino-acid datasets using site-homogeneous empirical models and a reduced taxon set. Those authors evoked the same kind of LBA artefact to explain what they considered as an artefactual result, as support for it disappeared in analyses including fewer sites but with fewer missing data [32]. These observations confirm that phylogenomic reconstruction can lead to artefacts, especially when the taxon sampling is reduced and model assumptions are violated [40]. When analysing large concatenations, use of best-fit models is required to account specifically for heterogeneities among genes and codons in the substitution process, and to alleviate the deleterious effects of substitution saturation. Similarly, we found that when using mixed models for analysing protein-coding gene concatenations, partitioning by codon position outperformed the widely used gene-partitioning scheme. The CAT-GTR + G mixture model nevertheless offers the most efficient solution to account explicitly for site heterogeneities in the substitution process as typically observed in phylogenomic datasets [41].

As illustrated by our results, statistical inconsistency is not restricted only to concatenation-based phylogenetic reconstruction methods. Although specifically designed to accommodate potential gene-tree discordances, species tree inference methods also seem to be sensitive to mis-specification of the substitution model used to infer gene trees. Indeed, the species tree obtained from the nucleotide dataset also strongly supported the artefactual grouping of crocodiles and turtles (Figure 3b). Therefore, in addition to their accounting for gene-tree heterogeneity, the use of the best-fitting substitution models seems to be equally important for these methods [42]. These results also indicate a potential problem of stochastic error in reconstructing gene trees for which only a limited number of sites is available, and the consequent effect on species tree inference. Ultimately, species trees can only be as good as the gene trees from which they are inferred.

Finally, it is worth noting that a recent analysis of miRNA phylogenetic distribution [31] supported a branching order (turtles + squamates) that was strongly rejected by our data. This is not the first instance of a conflict between miRNA and sequence-based phylogenetic studies, as shown by the case of acoels for instance [43,44]. Thus, our study suggests caution is needed when using miRNA markers in phylogenetic reconstructions, as they might not be as free from homoplasy as sometimes considered [45]. For example, secondary loss of multiple families of miRNAs have already been reported in tunicates [46]. Our results imply that the four miRNAs families exclusively shared by turtles and lizards [31] either have been lost secondarily in archosaurs, or have been independently recruited in turtle and lizard genomes. Upcoming reptile genomic data [47,48] should allow verification of these predictions.

### Consequences for interpreting character evolution in amniotes

The well-supported sister group relationship of the turtles to the archosaurs has important implications for the evolution of morphological, developmental, and ecological characters of amniotes. It implies, as previously proposed [15], that turtles experienced a secondary closure of the temporal fenestration, which therefore appears to be a derived character, rather than a reflection of the ancestral condition, as has long been assumed. In addition, because a basal position of turtles within reptiles is supported by the timing of events in organogenesis [49], our results suggest the occurrence of significant convergence in developmental timing characters, and advocate for the re-interpretation of sequence heterochrony data in the light of the phylogenetic position of turtles supported by our phylogenomic analyses. Finally, the assumption of an aquatic origin of turtles (the hypothesis that was brought forward due to the proposed sister group relationship between turtles and an extinct group of marine reptiles (Sauropterygia) and Lepidosauria [16]) also needs to be reconsidered. A recent study suggested, for example, that stem turtles could have occupied both terrestrial and aquatic habitats [50].

The proposed phylogeny of amniotes also provides a more solid background from which to investigate the evolution of the sex-determining systems and genomic characteristics of reptiles. Whereas mammals and birds have only genetic sex determination, non-avian reptiles have both genetic and temperature-dependent sex determination. Temperature-dependent sex determination also occurs in crocodilians, tuatara, and the majority of turtles, whereas it is less common in squamates [51,52]. Studies on this subject have relied on a traditional view of the vertebrate phylogeny, with turtles being basal to the other reptiles (including birds) (compare, for example Janzen & Krenz [51] with Janes *et al. *[53]). Although the phylogenetic scenario supported by our data would not change the main conclusion that temperature-dependent sex determination evolved multiple times within amniotes, it does provide a basis from which to further investigate the possible adaptive evolutionary value of the temperature-dependent sex determination in amniotes and the evolution of sex chromosomes.

Recently, Matsuda *et al. *[54] reported a high degree of conservation between the chromosomes of a turtle (*Pelodiscus sinensis*) and the common chicken, in accordance with an archosaurian affinity of turtles. These authors also suggested that although no specific sex chromosomes could be identified in the studied turtle, which has genetically determined sex, the ancestral avian Z sex chromosome has been conserved in the turtle genome. However, other features of the genome, such as its average genome size, GC content, and distribution of transposable elements show a marked similarity between turtles and crocodiles, to the exclusion of birds [29,55]. By rejecting the turtles plus crocodilians grouping, our analysis could possibly be interpreted as evidence for a parallel evolution of these genomic features in the two lineages, or, perhaps more plausibly, recent evolution of bird-specific features in the avian lineage.

### Models of molecular clock relaxation

In our molecular dating analyses, we found discrepancies between the results obtained using standard substitution models (LG + G and GTR + G) and the CAT-GTR + G mixture model with both amino acids and nucleotides. These differences probably stem from underlying differences in branch-length estimates between the two types of models, indicating the need to apply the most appropriate models of sequence evolution currently available for conducting molecular dating analyses [56]. Our results indicate that the CAT-GTR + G mixture model better accounts for the site-specific heterogeneities of our concatenated protein-coding gene datasets. Therefore, we consider that the divergence date estimates obtained under this model are the most reliable.

However, these small discrepancies between estimates obtained under different substitution models are almost negligible as compared with the large differences in estimates between the auto-correlated and uncorrelated models of rate change. In our case, the use of uncorrelated models generally led to unreasonably recent dating estimates for all nodes relative to the values reported in the literature [90]. These results seriously question the adequacy of the uncorrelated models of molecular clock relaxation parameters for estimating divergence times, at least with our dataset. Based on Bayes factor comparisons, Lepage *et al. *[56] showed that auto-correlated models provide a significantly better fit than the uncorrelated gamma model, especially for large phylogenomic datasets. These results were recently confirmed in an empirical phylogenomic study focusing on the estimation of arthropod divergence times, for which the assumption of rate autocorrelation seemed to be the most realistic way of modelling evolutionary rate variations across the tree [56,57]. For these reasons, we consider the results from the auto-correlated relaxed clock analyses under the CAT-GTR + G substitution model as our most reliable dating estimates (Table 3; Figure 4).

### Paleontological implications

Our auto-correlated relaxed clock analyses based on the CAT-GTR + G model support a divergence between turtles and Archosauria around 255 Mya (274-233 Mya), which is in agreement with the estimates recently reported by Shen *et al. *[30]. The dating obtained for other nodes also seems to be mostly compatible with current knowledge. For example, the Testudinoidea MRCA corresponding to the divergence between *Emys *and *Chelonoidis *is estimated at a mean of 91 Mya (range 131 to 54 Mya), which is comparable with that obtained by Lourenço *et al. *[58]. Exceptions concern squamates and crocodilians, for which our estimates indicated a more recent time than generally reported (Table 3). We note that the confidence intervals are relatively large, however, as would be expected for such indirect estimates, in which dates are estimated jointly with the process of substitution-change variations over time [59].

The single major difference between our estimates and the previously published divergence dates concern the MRCA of living turtles. This is a controversial issue in the paleontological literature, with proposed ages of divergence between the two main turtle lineages (Pleurodira and Cryptodira) varying from the Upper Triassic to the Upper Jurassic. This debate is mostly due to the rarity and the need for better characterization of turtle fossils older than 160 Mya [60]. Our analyses suggest that the MRCA of Chelonia (Pleurodira plus Cryptodira) is on average 157 Mya (range 207 to 104 Mya), taking the average mean and extremes of 95% credibility intervals for the CAT-GTR + G model amino-acid and nucleotide results. This means that an Upper Triassic origin (229 to 200 Mya) of extant turtle lineages is rejected, and that although a Lower Jurassic origin (200 to 176 Mya) is still possible, it seems unlikely. Remarkably, a similar conclusion was reached in a recent study using a fully independent molecular dataset, which only included turtle sequences and within-turtle fossil calibrations [58]. Our 95% credibility intervals for the turtle ancestral node (185 to 104 Mya with amino acids, and 207 to 120 Mya with nucleotides) are nevertheless wider and probably less realistic in including the Lower Cretaceous (146 to 97 Mya). However, taken together, these two analyses begin to suggest that the origin of Chelonia may be in the Middle or Upper Jurassic (176 to 146 Mya) or later. If so, two controversial fossils, *Proterochersis *and *Kayentachelys*, attributed respectively to the Cryptodira and Pleurodira clades, would be currently misplaced on the turtle lineage. These placements, proposed by Gaffney [61], have been a subject of intense debate [62,63] (references therein). This would also have important implications for molecular clock analyses, because these fossils are usually used for calibrating both turtle [64] and amniote [30] trees. Considering this, it may be prudent to consider these fossils Testudines *incerte sedis *until additional data can be obtained to confirm their phylogenetic placement.

## Conclusions

As already shown in cases of other difficult phylogenetic questions, we found analyses of phylogenomic data to be useful in resolving the uncertain placement of turtles within the phylogeny of amniotes. In fact, our analyses show that the hypothesis of a sister group relationship between turtles and crocodilians is most likely a phylogenetic reconstruction artefact related to substitution saturation. When this artefact is taken into account by using the best models of sequence evolution currently available, we found strong support in all cases for identifying turtles as a sister group to Archosauria, to the exclusion of any alternative phylogenetic hypothesis. Our results confirming turtles as derived diapsids have important implications for understanding the evolution of morphological characters and for interpreting developmental and genomic data in amniotes. Finally, our results shed light on another debated topic by contesting the ancient Lower Jurassic origin of the two main extant lineages of turtles. Indeed, our molecular dating places the MRCA of living turtles in the Upper Jurassic period, a more recent estimate than previously reported, and one that questions the interpretation of controversial Lower Jurassic fossils considered as 'crown turtles'.

## Methods

### Transcriptome data acquisition

Blood samples were obtained from four species of turtle for which genomic data were not already available: *Phrynops hilarii*, *Caretta caretta*, *Chelonoidis nigra*, and *Emys orbicularis*, representing the two suborders Pleurodira and Cryptodira. We also took a jaw sample from a crocodilian (*Caiman crocodilus*) and a liver sample from a lacertid lizard (*Podarcis *sp.). A jaw sample from a lungfish (*Protopterus annectens*) was used to provide an outgroup for the phylogenetic analyses.

RNA was extracted and checked for quality and quantity in accordance with previously described protocols [65,66]. Transcriptome sequencing using the 454 GS-FLX Titanium platform (454 Life Sciences, Branford, Connecticut, USA) was performed by GATC Biotech (Konstanz, Germany). We also retrieved 454 sequencing reads from the National Center for Biotechnology Information (NCBI) Sequence Read Archive (SRA) for two additional reptile species: a snake (*Python molurus bivittatus*; SRX072633, SRX072634, SRX057862, and SRX018167 [67,68]) and an alligator (*Alligator mississippiensis*; SRX012365 [69]). All raw sequencing reads were cleaned from sequencing adaptors and then assembled into *de novo *contigs for each of the nine species using either CAP3 [70] or PCAP [71] assembly software. The basic statistics on raw reads and contig assemblies are indicated in Table 4.

**Table 4:** Basic sequencing and assembly statistics for the seven newly sequenced and the two additional transcriptomes included in this study.

Species	Number of 454 reads	Mean read length after cleaning	Number of contigs	Mean contig length
Phrynops hilarii*	454,711	303	45,554	548
Caretta caretta*	377,038	250	34,440	450
Chelonoidis nigra*	426,721	270	55,272	481
Emys orbicularis*	522,009	357	39,166	588
*Alligator mississippiensis*	436,439	266	29,309	400
Caiman crocodilus*	343,080	217	22,025	378
*Podarcis *sp.*	303,076	198	20,756	357
*Python molurus bivittatus*	950,283	232	36,334	446
Protopterus annectens*	666,034	231	37,810	469

*Newly sequenced in this study.

### Data assembly

For constructing our phylogenomic dataset, we designed an analytical pipeline aiming at conservatively selecting a set of single-copy orthologous genes that would also minimize the amount of missing data in the assembled dataset (see Additional file 3). We relied on the EnsemblCompara phylogenetic assessment of orthology [72] by downloading the 7,943 (Ensembl, release 60) coding sequences (CDSs) of the 1:1 orthologous genes shared by *Homo sapiens*, *Monodelphis domestica*, *Gallus gallus*, and *Taeniopygia guttata*. We then added all CDSs that are predicted to be 1:1 orthologous genes between *H. sapiens *and *Ornithorhynchus anatinus*, *H. sapiens *and *Anolis carolinensis*, and *H. sapiens *and *Xenopus tropicalis*. This resulted in 4,305 1:1 orthologous CDSs that are shared by these seven core species. A best reciprocal hit (BRH) strategy was then used to identify 1:1 orthologs from the contigs of the nine assembled transcriptomes. We performed tBLASTx searches, using the 4,305 CDSs of *G. gallus *against each contig set (parameters: length > 100 nucleotides, score > 100, e-value < 1e^-100^, and identity > 50%). Another BLAST search was then performed for each matching contig against the full CDS set of *G. gallus *(17,934 genes), and only contigs with a significant BRH on exactly the same CDS were conserved. This BRH step led to 2,118 1:1 orthologous CDSs, for which at least one contig from the nine transcriptomes was added to the initial set of seven species. These datasets were then filtered taxonomically using the PhyloExplorer software [73], to keep only the 367 datasets that contained at least one turtle and one crocodile.

The resulting 367 CDS multiFASTA files were then aligned with MACSE [74] using the next-generation sequencing default settings option. This allowed us to align the newly assembled contigs against the seven reference CDSs from Ensembl, while respecting the coding frame by inserting frameshifts and stop codons in assembled contigs where needed. The 367 nucleotide alignments were then manually curated and trimmed, based on MASCE annotations of frameshifting events. Datasets in which turtle and crocodile sequences did not overlap were excluded. ML trees were then inferred from the remaining 331 alignments using PHYML (version 3.0) [75] with SPR moves on a BIONJ starting tree under the GTR + G8 model. We next excluded genes for which amniotes were not monophyletic as they are likely to correspond to orthology assessment problems of the *Xenopus *and/or *Protopterus *sequences used as outgroups. Ambiguously aligned codons were filtered out from the resulting 260 alignments by using Gblocks [76] with default parameters. After excluding the datasets containing less than 300 nucleotide sites, the concatenation of the final 248 CDS datasets represented a total of 187,026 nucleotide sites (62,342 amino-acid sites) for 16 taxa, with only 35% missing data. These two final datasets have been deposited in the Dryad digital repository [77]. A table indicating the chicken Ensembl gene identification numbers (IDs) and official gene names of the 248 genes used is provided (see Additional file 4).

### Phylogenetic analyses

Phylogenetic analyses were performed using both ML and Bayesian reconstruction methods on the nucleotide and amino-acid datasets. ML analyses of the different concatenations (amino acids, all nucleotide sites, codon positions 1 + 2, and codon positions 3) were first conducted using RAxML (version 7.2.8) [78] using a single LG + F + G model for amino acids, and a single GTR + G model for nucleotide datasets. We also performed ML searches under mixed models partitioned by gene (248 partitions) and codon position (3 partitions) using the same LG + F + G and GTR + G models for each amino-acid and nucleotide partitions, respectively. In mixed-model analyses, branch lengths were optimized individually per partition. ML bootstrap values were computed by repeating the original ML heuristic search on 100 bootstrap pseudoreplicates for each dataset. The AU statistical test for comparing alternative topologies was computed using CONSEL [79] from site-wise log-likelihood values estimated by RAxML for four *a priori *competing phylogenetic hypotheses for the position of turtles.

Bayesian phylogenetic inferences were conducted using MrBayes (version 3.1.2) [80] using a single WAG + G model for amino acids and a single GTR + G model for nucleotide datasets. We also applied the Bayesian approach using a mixed model partitioned by codon position (three partitions) with a GTR + G model for each nucleotide partition. In this mixed-model analysis, all model parameters including branch lengths were unlinked between partitions. All Bayesian analyses were computed using four incrementally heated Metropolis-coupled Markov chain Monte Carlo (MCMCMC) run for 1,000,000 generations, with trees and associated model parameters sampled every 100 generations. The initial 1000 sampled trees (10%) were discarded as the burn-in, and the 50% majority-rule Bayesian consensus tree and associated clade PPs were computed from the remaining 9000 trees.

We also performed Bayesian phylogenetic analyses under the site-heterogeneous CAT-GTR + G4 mixture model [81] on both amino-acid and nucleotide datasets using PhyloBayes (version 3.3b) [82]. To avoid potential biases associated with a large proportion of invariable sites in estimating the site-heterogeneous CAT profiles, analyses of codon positions 1 + 2 and codon position 3 were conducted with constant sites excluded (-dc option). In each individual analysis, two independent MCMC chains starting from a random tree were run for 20,000 cycles, with trees and associated model parameters being sampled every 10 cycles until 2,000 trees were sampled. The initial 200 trees (10%) sampled in each MCMC run were discarded as the burn-in period. The 50% majority-rule Bayesian consensus tree and the associated PP_CAT _were then computed from the remaining 3,600 trees combined from the two independent runs.

Finally, to account for potential discordances between gene trees and the species tree, we used the pseudo-ML approach implemented in the MP-EST program [83]. The species tree was inferred under the coalescent model from the 248 individual ML gene trees obtained by PHYML with SPR moves on a BIONJ starting tree under the GTR + G8 model for nucleotides and the LG + G8 model for amino acids. The reliability of the species tree inference was assessed using a nonparametric bootstrapping procedure resampling sites within individual genes [84]. PHYML, using the same settings as described above, was first used to infer 100 ML bootstrap trees from each individual gene dataset, then the initial MP-EST species tree inference was repeated 100 times from each of the 248 individual bootstrap gene-tree replicates. Bootstrap percentages were finally obtained by computing the 50% majority-rule consensus tree from the resulting 100 bootstrap MP-EST species trees.

### Molecular dating analyses

Dates of divergence between amniotes were estimated using the Bayesian relaxed molecular clock approaches implemented in PhyloBayes, MCMCTree from the PAML (version 4.5) package [85], and BEAST (version 1.7) [86], using the Bayesian consensus topology obtained from nucleotides (Figure 1b). With PhyloBayes, both amino-acid and nucleotide datasets were analysed under the CAT + GTR + G4 mixture model, and under the standard LG + G and GTR + G models, respectively. Besides PhyloBayes, two alternative molecular dating programs were used to replicate the analyses under distinct implementations of the Bayesian exploration of clock-relaxed models. The standard WAG + G and GTR + G models were used in MCMCTree and BEAST for analysing amino-acid and nucleotide datasets, respectively. In MCMCTree, we used the ML approximation by first calculating the ML estimates of the branch lengths, the gradient vector and Hessian matrix, using the BaseML and CodeML programs of PAML. Despite the fact that auto-correlated models of clock relaxation have been shown to provide a significantly better fit than uncorrelated models on phylogenomic datasets [56,57], all analyses were conducted under both models of molecular clock relaxation for comparison purposes. As BEAST implements only uncorrelated relaxed clock models, we used the uncorrelated log_normal _model.

Six fossil calibrations compatible with our tree were selected from Benton *et al. *[87]: (1) *Xenopus*/*Homo *(350 Myr to 330 Myr), (2) *Gallus*/*Homo *(330 to 312), (3) *Anolis*/*Gallus *(300 to 256), (4) *Ornithorhynchus*/*Homo *(191 to 163), (5) *Monodelphis*/*Homo *(171 to 124), and (6) *Gallus*/*Taeniopygia *(87 to 66). These calibration constraints were used with soft bounds [88] under a birth-death prior in PhyloBayes and MCMCTree, because this strategy has been shown to provide the best compromise for dating estimates [89]. The prior on the root age corresponding to the *Protopterus*/*Homo *split was set at 419 to 408 Myr [90]. In BEAST, we used normal distributions with 95% confidence intervals covering these constraints as calibration priors with a birth-death process on the tree.

In PhyloBayes, all calculations were conducted by running two independent MCMC chains for 20,000 cycles, sampling posterior rates and dates every 10 cycles until 2000 points were collected. Posterior estimates of divergence dates were then computed from the last 1800 samples of each chain after accounting for the initial burn-in period (10%). In MCMCTree, two independent MCMC chains were run with the following parameters: burn in = 1,000,000; sampling frequency = 100; number of samples = 10,000,000. The first 1,000,000 iterations were thus discarded as burn-in, and then the MCMC was run for 100,000,000 iterations, sampling every 100 iterations. The 10,000,000 samples were then summarized to estimate mean divergence date and associated 95% credibility intervals. Finally, BEAST was set up using a single MCMC run for 5,000,000 and 10,000,000 generations for analysing the amino-acid and nucleotide datasets, respectively. Each chain was sampled every 10,000 generations to generate 5,000 and 10,000 samples, of which the first 10% were excluded as the burn-in before computing the mean divergence time estimates and associated 95% credibility intervals.

## Competing interests

The authors declare that they have no competing interests.

## Authors' contributions

NG, FD and YC conceived and designed the study. YC and FD collected biological material. YC carried out RNA extractions and coordinated transcriptomes sequencing. VC performed sequence database construction, contigs assembly, and BRH calculations. FD and YC constructed the phylogenomic dataset. FD conducted the phylogenetic and dating analyses. YC, NG and FD wrote the manuscript. All authors read and approved the final manuscript.

## Supplementary Material

Additional file 1 **Table S1: Detailed results of Bayesian relaxed molecular clock analyses obtained under different uncorrelated models for the eight unconstrained nodes**. Click here for file

Additional file 2 **Figure S1: Maximum likelihood analyses of the nucleotide dataset**. ML phylograms with branch lengths obtained using RAxML with a single concatenated GTR + G model for analysing **(a) **the complete nucleotide dataset, **(b) **codon positions 1 + 2, and **(c) **third codon positions only. Click here for file

Additional file 3 **Figure S2: Analytical pipeline used for assembling the phylogenomic dataset**. Click here for file

Additional file 4 **Table S2: Chicken Ensembl gene IDs and official gene names of the 248 markers used in this study**. Click here for file
