# Supplementary material for: Phylogenomic analyses support the position of turtles as the sister group of birds and crocodiles (Archosauria)
Source: BMC Biol. 2012 Jul 27;10:65. doi: 10.1186/1741-7007-10-65 (PMC3473239; doi:10.1186/1741-7007-10-65)
Supplement: Additional file 1 — Table S1: Detailed results of Bayesian relaxed molecular clock analyses obtained under different uncorrelated models for the eight unconstrained nodes. [file 1741-7007-10-65-S1.PDF]

Table S1 - Detailed results of Bayesian relaxed molecular clock analyses obtained under different uncorrelated models for the eight unconstrained nodes (Mean [95% Credibility interval]).

	AA				NT				TimeTree[1]
	BEAST WAG+G	MCMCTree WAG+G	Phylobayes LG+G	Phylobayes CAT-GTR+G	BEAST GTR+G	MCMCTree GTR+G	Phylobayes GTR+G	Phylobayes CAT-GTR+G	Mean / Median
**1. Turtles / Archosaurs**	204 [153-262]	193 [152-238]	190 [137-253]	200 [128-270]	193 [168-238]	183 [142-236]	183 [119-263]	200 [116-284]	244 / 265
**2. Archosaurs MRCA**	137 [110-171]	160 [121-204]	155 [112-213]	159 [104-238]	172 [150-218]	161 [120-212]	153 [100-231]	162 [98-249]	238 / 245
**3. Turtles MRCA**	64 [27-99]	67 [42-111]	71 [38-151]	68 [31-167]	78 [56-123]	52 [30-90]	68 [23-171]	81 [17-228]	207 / 211
**4. *Caretta* / *Emys+Cheloinidis***	34 [13-55]	36 [21-59]	37 [18-86]	34 [14-100]	36 [20-53]	27 [15-46]	35 [11-131]	32 [7-128]	97 / 99
**5. *Emys* / *Chelonoidis***	27 [7-47]	29 [16-50]	29 [13-73]	25 [8-83]	27 [10-42]	20 [9-36]	21 [5-79]	23 [3-130]	70 / 70
**6. *Caiman* / *Alligator***	39 [13-67]	43 [21-78]	45 [19-96]	42 [13-104]	20 [6-38]	24 [10-48]	25 [9-63]	24 [4-85]	70 / 72
**7. *Podarcis* / *Python+Anolis***	143 [100-204]	159 [101-202]	154 [33-208]	142 [58-206]	96 [76-118]	155 [83-200]	151 [84-204]	120 [47-217]	190 / 178
**8. *Python* / *Anolis***	118 [82-161]	141 [82-182]	135 [68-190]	127 [44-195]	75 [65-91]	139 [66-182]	133 [67-183]	97 [26-190]	171 / 163
