# Supplementary figures and images for: Phylogenomic analyses support the position of turtles as the sister group of birds and crocodiles (Archosauria)
Source: BMC Biol. 2012 Jul 27;10:65. doi: 10.1186/1741-7007-10-65 (PMC3473239; doi:10.1186/1741-7007-10-65)

a

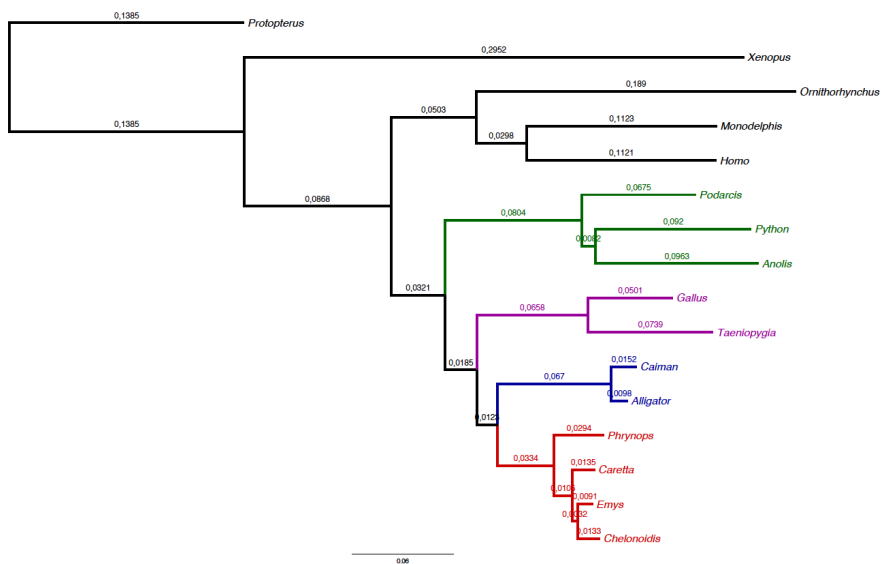

b

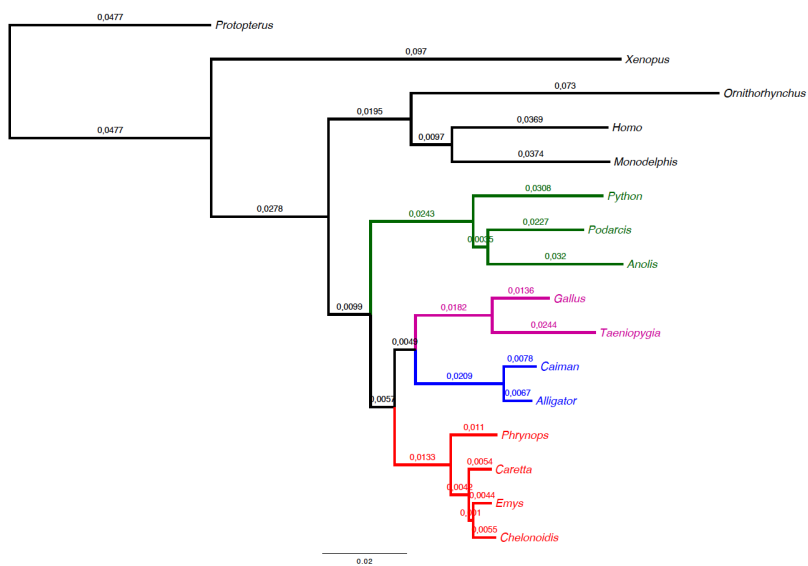

c

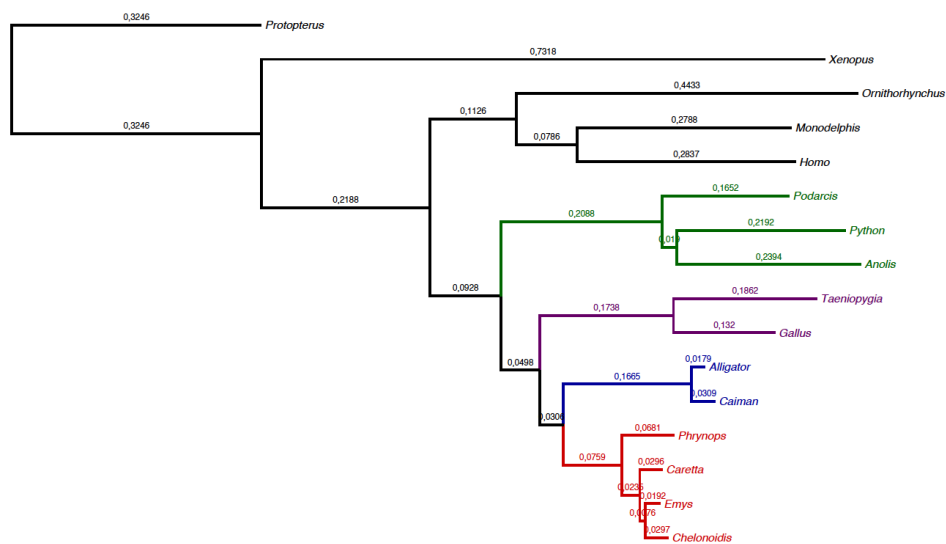

Supplement: Additional file 2 — Figure S1: Maximum likelihood analyses of the nucleotide dataset. ML phylograms with branch lengths obtained using RAxML with a single concatenated GTR + G model for analysing (a) the complete nucleotide dataset, (b) codon positions 1 + 2, and (c) third codon positions only. [file 1741-7007-10-65-S2.PDF]

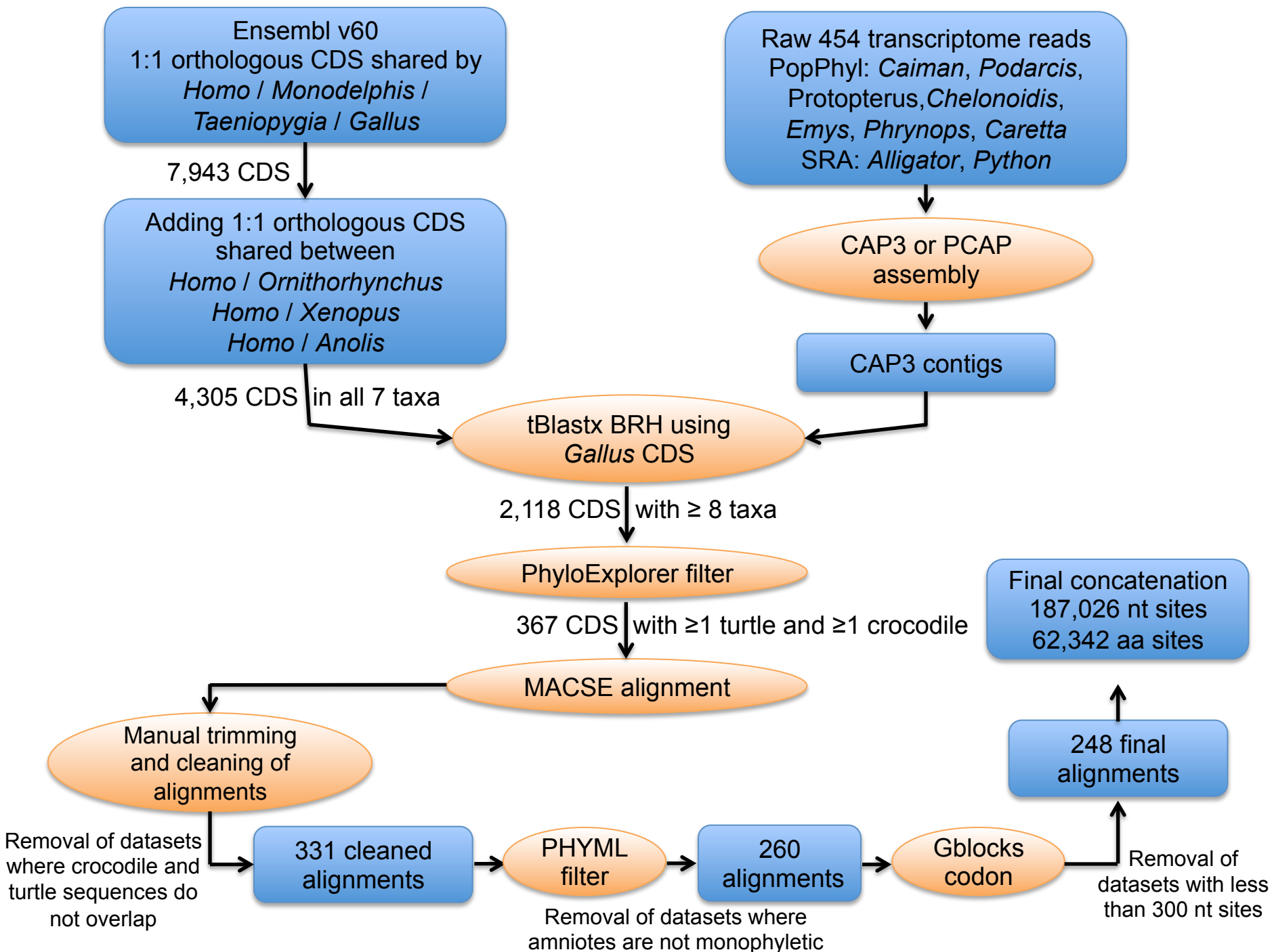

Supplement: Additional file 3 — Figure S2: Analytical pipeline used for assembling the phylogenomic dataset. [file 1741-7007-10-65-S3.PDF]
